# Photometric detection at 7.7 μm of a galaxy beyond redshift 14 with JWST/MIRI

**DOI:** 10.1038/s41550-025-02503-z

**Published:** 2025-03-07

**Authors:** Jakob M. Helton, George H. Rieke, Stacey Alberts, Zihao Wu, Daniel J. Eisenstein, Kevin N. Hainline, Stefano Carniani, Zhiyuan Ji, William M. Baker, Rachana Bhatawdekar, Andrew J. Bunker, Phillip A. Cargile, Stéphane Charlot, Jacopo Chevallard, Francesco D’Eugenio, Eiichi Egami, Benjamin D. Johnson, Gareth C. Jones, Jianwei Lyu, Roberto Maiolino, Pablo G. Pérez-González, Marcia J. Rieke, Brant Robertson, Aayush Saxena, Jan Scholtz, Irene Shivaei, Fengwu Sun, Sandro Tacchella, Lily Whitler, Christina C. Williams, Christopher N. A. Willmer, Chris Willott, Joris Witstok, Yongda Zhu

**Affiliations:** 1 Steward Observatory, University of Arizona, Tucson, AZ USA; 2 Center for Astrophysics, Harvard & Smithsonian, Cambridge, MA USA; 3 Scuola Normale Superiore, Pisa, Italy; 4 Kavli Institute for Cosmology, University of Cambridge, Cambridge, UK; 5 Cavendish Laboratory, University of Cambridge, Cambridge, UK; 6 European Space Astronomy Centre (ESAC), European Space Agency (ESA), Madrid, Spain; 7 Department of Physics, University of Oxford, Oxford, UK; 8 Institut d’Astrophysique de Paris, Sorbonne Université, CNRS, UMR 7095, Paris, France; 9 Department of Physics and Astronomy, University College London, London, UK; 10 Centro de Astrobiologia (CAB), CSIC-INTA, Madrid, Spain; 11 Department of Astronomy and Astrophysics, University of California, Santa Cruz, CA USA; 12 NSF’s National Optical–Infrared Astronomy Research Laboratory, Tucson, AZ USA; 13 NRC Herzberg, Victoria, British Columbia Canada

**Keywords:** Galaxies and clusters, Early universe

## Abstract

The James Webb Space Telescope (JWST) has spectroscopically confirmed numerous galaxies at *z* > 10. While weak rest-frame ultraviolet emission lines have only been seen in a handful of sources, the stronger rest-frame optical emission lines are highly diagnostic and accessible at mid-infrared wavelengths with the Mid-Infrared Instrument (MIRI) of JWST. We report the photometric detection of the distant spectroscopically confirmed galaxy JADES-GS-z14-0 at $z=14.3{2}_{-0.20}^{+0.08}$ with MIRI at 7.7 μm. The most plausible solution for the stellar-population properties is that this galaxy contains half a billion solar masses in stars with a strong burst of star formation in the most recent few million years. For this model, at least one-third of the flux at 7.7 μm originates from the rest-frame optical emission lines Hβ and/or [O iii]*λλ*4959, 5007. The inferred properties of JADES-GS-z14-0 suggest rapid mass assembly and metal enrichment during the earliest phases of galaxy formation. This work demonstrates the unique power of mid-infrared observations in understanding galaxies at the redshift frontier.

## Abstract (web summary)

The authors report the photometric detection of the distant spectroscopically confirmed galaxy JADES-GS-z14-0 at *z* > 14 with JWST/MIRI. The inferred properties suggest rapid mass assembly and metal enrichment during the earliest phases of galaxy formation.

## Main

With the launch of the James Webb Space Telescope (JWST), extragalactic astronomy fundamentally changed. The Near Infrared Camera (NIRCam) moved the photometric redshift frontier from *z* ≈ 10 to *z* ≈ 14–16 (see, for example, refs. ^1–6^), while the Near Infrared Spectrograph (NIRSpec) pushed the spectroscopic redshift frontier from *z* ≈ 8 to *z* ≈ 12–14 (see, for example, refs. ^7–10^). Crucially, JWST discovered an early period of galaxy formation that was more vigorous than expected, with a sizable population of luminous galaxies and supermassive black holes less than a billion years after the Big Bang.

A companion paper reports the spectroscopic confirmation of JADES-GS-z14-0 at redshift $z=14.3{2}_{-0.20}^{+0.08}$, which makes it the most distant galaxy with a spectroscopically confirmed redshift^11^. This galaxy is remarkably luminous, with a rest-frame UV absolute magnitude *M*_UV_ ≈ −20.81 ± 0.16, which may require a reassessment of ideas about early galaxy formation, suggesting a slow decline in the number density of galaxies at *z* > 12, with increasing efficiency of galaxy formation in halos at higher redshifts^5^. The rest-frame UV continuum slope *β*_UV_ ≈ −2.20 ± 0.07 is relatively red for a very young stellar population, suggesting that the UV emission is affected by a small amount of dust attenuation. The full-width at half-maximum (FWHM) of the intrinsic rest-frame UV light profile is 0.16 ± 0.01 arcsec (corresponding to a deconvolved half-light radius of 260 ± 20 pc). Given its spatial extent, the rest-frame UV emission appears not to be dominated by emission from an active galactic nucleus. The properties of JADES-GS-z14-0 add to the evidence that a population of luminous and massive galaxies was already in place less than 300 Myr after the Big Bang, with number densities more than ten times higher than extrapolations based on pre-JWST observations, as demonstrated in ref. ^5^.

The rest-frame optical nebular emission lines are one of the primary means to characterize the physical conditions in galaxies. However, the redshift of JADES-GS-z14-0 has moved these lines into the wavelength coverage of the Mid-Infrared Instrument (MIRI), beyond the wavelength coverage of NIRCam and NIRSpec. The superb performance of JWST^12^, alongside the remarkable brightness of the most extreme high-redshift galaxies, will allow MIRI to explore the rest-frame optical regime and provide important insights into the nature of the earliest galaxies. MIRI has been used at the redshift frontier with the recent spectroscopic identification of the rest-frame optical emission lines [O iii]*λλ*4959, 5007 and Hα in the galaxy GHZ2/GLASS-z12 at *z* = 12.33 ± 0.02 (ref. ^13^). These results highlight the power of combining observations from NIRCam, NIRSpec and MIRI to understand the properties of the very first galaxies.

In this work, we present the robust photometric detection of JADES-GS-z14-0 with MIRI at an observed wavelength of *λ*_obs_ ≈ 7.7 μm, corresponding to rest-frame wavelengths of *λ*_rest_ ≈ 4,400–5,700 Å. These ultradeep observations with MIRI/F770W provide more information about the nature of this remarkable galaxy, and are among the deepest mid-infrared integrations to date, with an on-source integration time of *t*_obs_ ≈ 23.8 h for JADES-GS-z14-0.

**Fig. 1:**
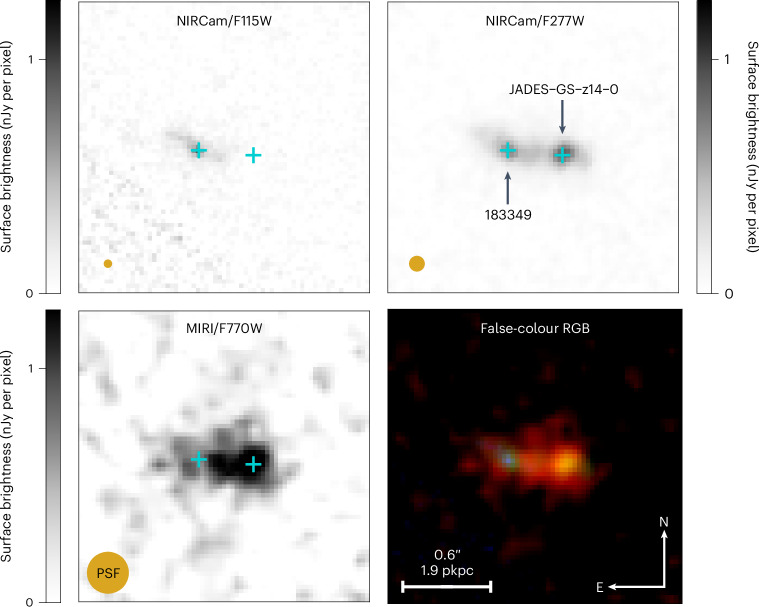
A distant galaxy spectroscopically confirmed by JADES. This galaxy was initially selected from ultradeep NIRCam and MIRI imaging with JWST (F770W–F277W–F115W shown as an RGB false-colour mosaic in the lower right). It was targeted for NIRSpec micro shutter array follow-up observations and has been spectroscopically confirmed at redshift $z=14.3{2}_{-0.20}^{+0.08}$^11^. JADES-GS-z14-0 is to the right and the foreground galaxy NIRCam ID 183349 to the left. The apparent colour of JADES-GS-z14-0 is caused by the absorption of the NIRCam/F115W flux by the intervening IGM and the rest-frame optical nebular emission-line excess in MIRI/F770W relative to NIRCam/F277W. A scale bar of 0.6" is provided which corresponds to roughly 1.9 physical kpc (pkpc) at the observed redshift (*z* = 14.32).

Figure 1 presents in the lower right the JWST F770W–F277W–F115W false-colour image for JADES-GS-z14-0. The apparent colour of this galaxy is caused by (1) the complete absorption of emission by the intergalactic medium (IGM) in the F115W filter and (2) the excess rest-frame optical emission in the F770W filter relative to the rest-frame UV emission in the F277W filter. JADES-GS-z14-0 is close in projection to a foreground galaxy at a separation of roughly 0.4 arcsec to the east, which we refer to as NIRCam ID 183349 (ref. ^11^). The lensing magnification caused by 183349 and another low-redshift neighbouring object (roughly 2.2 arcsec to the south) is estimated to be small, with a lensing magnification factor of *μ* = 1.2 (ref. ^11^). All of the analyses and results presented here have been corrected for this magnification factor.

**Extended Data Fig. 1:**
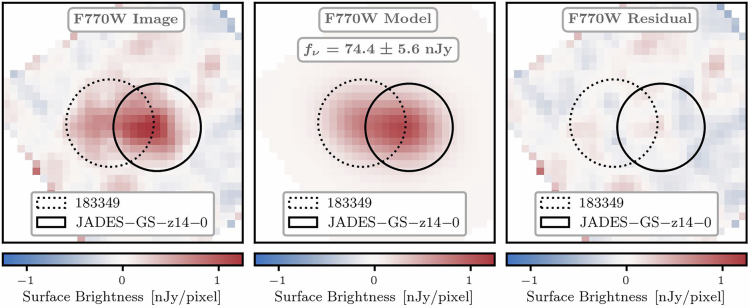
An example of the model fitting photometry for JADES-GS-z14-0. The individual exposures from the MIRI/F770W imaging are modeled assuming an extended morphology. These images are dominated by the diffraction width of the F770W PSF (FWHM = 0.269 arcseconds), which is nearly twice the measured half-light angular diameter with NIRCam (FWHM = 0.145 arcseconds). We measure photometry by fitting surface brightness profiles to the flux image of the object and its neighbors (left panel), allowing us to construct accurate models (middle panel) which leave only slight residuals (right panel) relative to the data. The thumbnails are 2” × 2” which corresponds to roughly 6.5 pkpc × 6.5 pkpc at the observed redshift (z = 14.32). We note that the mean stacked flux image of the individual exposures is provided in the left panel for illustrative purposes, rather than the final image mosaic from Fig. 1 and Extended Data Figure 2.

**Extended Data Fig. 2:**
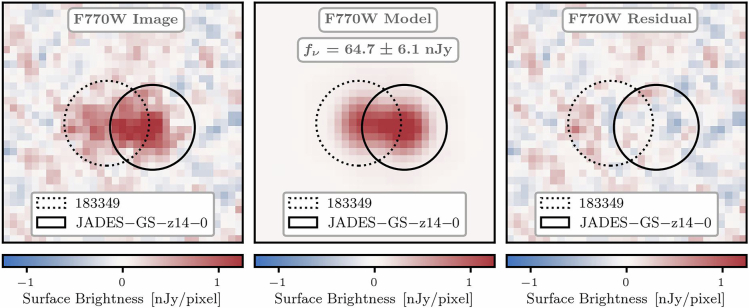
An example of the PSF photometry for JADES-GS-z14-0. The final image mosaic for the MIRI/F770W imaging is modeled assuming a point source morphology for both galaxies. We measure photometry by fitting surface brightness profiles to the flux image of the object and its neighbors (left panel), allowing us to construct an accurate model of the image (middle panel) that leaves only slight residuals (right panel) relative to the data. The thumbnails are 2” × 2” which corresponds to roughly 6.5 pkpc × 6.5 pkpc at the observed redshift.

Interpreting the ultradeep MIRI observations in the context of the ultradeep NIRCam observations requires measurement of the flux density in MIRI relative to the flux density in one of the long-wavelength NIRCam filters. The FWHMs of the F770W and F444W point spread functions (PSFs) are 0.269 arcsec and 0.145 arcsec, respectively, which makes separating JADES-GS-z14-0 from 183349 challenging, but essential. To meet this challenge, we measure photometry by performing model fitting on the individual exposures before mosaicing, illustrated by Extended Data Fig. 1. JADES-GS-z14-0 and 183349 are fitted simultaneously in all of the NIRCam and MIRI exposures with ForcePho (B.D.J. et al., in preparation) and GALFIT^14,15^. We model the sources with Sérsic light profiles. An image of the difference between the observations and our model shows only small residuals, demonstrating the validity of the modelling. We measure the F444W flux density to be *f*_F444W_ = 46.9 ± 0.6 nJy and the F770W flux density to be *f*_F770W_ = 74.4 ± 5.6 nJy for JADES-GS-z14-0. The measurements correspond to an excess flux of Δ*f* = 27.5 ± 5.6 nJy in F770W with respect to F444W. We report these measurements in Table 1. Simple PSF photometry confirms these results (see Extended Data Fig. 2 for detailed results). The quoted photometric uncertainties indicate the signal-to-noise ratios (*S*/*N*) but are probably underestimates of the true errors, since we do not account for systematic uncertainties related to, for example, photometric calibration, background subtraction and/or parametric assumptions for the intrinsic light profiles of JADES-GS-z14-0 and 183349.

**Table 1:** Photometry for JADES-GS-z14-0

Instrument/Filter	Model fitting (nJy)
NIRCam/F090W	−2.1 ± 0.6
NIRCam/F115W	−0.8 ± 0.4
NIRCam/F150W	1.2 ± 0.5
NIRCam/F162M	−1.5 ± 0.9
NIRCam/F182M	13.9 ± 0.4
NIRCam/F200W	34.8 ± 0.5
NIRCam/F210M	46.5 ± 0.6
NIRCam/F250M	47.2 ± 0.5
NIRCam/F277W	55.1 ± 0.6
NIRCam/F300M	49.8 ± 0.5
NIRCam/F335M	43.4 ± 0.5
NIRCam/F356W	47.3 ± 0.5
NIRCam/F410M	46.1 ± 0.8
NIRCam/F444W	46.9 ± 0.6
MIRI/F770W	74.4 ± 5.6

Columns: (1) instrument and filter combinations and (2) fiducial model-fitting photometry assuming an extended morphology.

**Extended Data Fig. 3:**
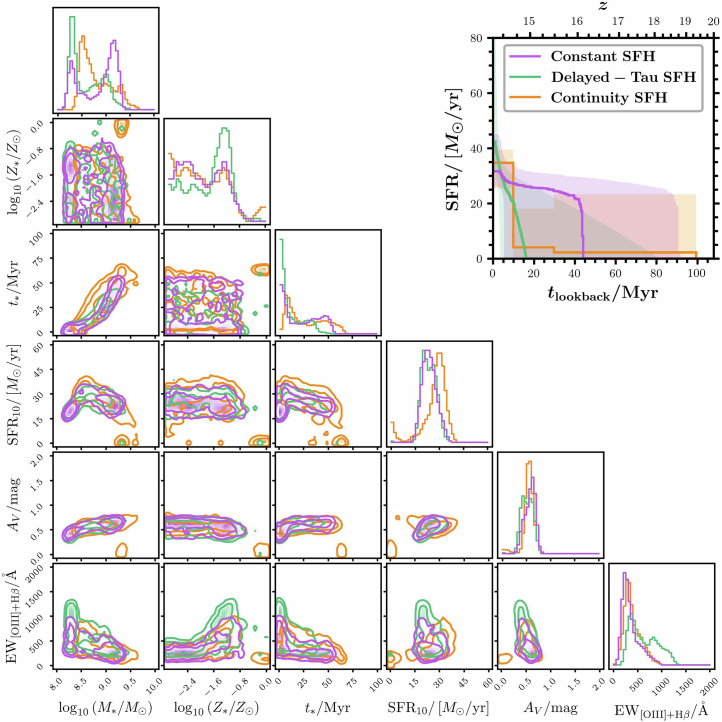
Constraints on joint posterior distributions and star formation histories (SFHs) with BAGPIPES. Various SED models with different SFH assumptions (constant in purple, delayed-tau in green, and continuity in orange) are implemented using BAGPIPES and the measured model fitting photometry. In the lower left, the joint posterior distributions are shown for stellar mass (M_*_), stellar metallicity (Z_*_), mass-weighted stellar age (t*), star-formation rate averaged over the previous 10 million years (SFR_10_), diffuse dust attenuation as measured in the V-band (A_V_), and rest-frame equivalent width for the nebular emission lines H*β* and [OIII]*λλ*4959, 5007 (EW_[OIII]+H*β*_). In the upper right, the derived SFHs are shown. The medians of the derived SFHs are the colored lines, while the 1*σ* confidence intervals are the shaded regions. This figure highlights degeneracies between some of the inferred physical parameters.

To interpret the source of the excess flux in F770W relative to F444W, we model the spectral energy distribution (SED) of JADES-GS-z14-0 using two Bayesian fitting codes: BAGPIPES (Bayesian Analysis of Galaxies for Physical Inference and Parameter Estimation)^16^ and Prospector^17^. Generally, we find two types of solution for the stellar-population properties. To explain the red rest-frame UV continuum slope, BAGPIPES prefers solutions with relatively young stellar populations and non-zero diffuse dust attenuation, while Prospector prefers solutions with older stellar populations but roughly zero diffuse dust attenuation. The Prospector models contain a strong Balmer break (Extended Data Fig. 3), predicting stellar masses that are nearly an order of magnitude larger than the BAGPIPES models (Extended Data Table 1), rivalling the maximum expected halo mass for galaxies at these redshifts. Additionally, the Prospector models have the bulk of their stars forming at *z* ≈ 18–20 (corresponding to median mass-weighted stellar ages of *t*_★_ ≈ 80–100 Myr), with no recent star formation, which exacerbates concerns about the predicted stellar mass. The lack of recent star formation would be unexpected given the predicted burstiness of star formation in early galaxies (see, for example, ref. ^18^). Reference ^19^ shows that galaxies at the redshift frontier should have stellar masses, stellar ages and diffuse dust attenuations that are consistent with the predictions from the BAGPIPES models (see discussion in Methods). Furthermore, ref. ^20^ shows that the strength of the Balmer break is reduced substantially with a top-heavy initial mass function (IMF), as is likely for JADES-GS-z14-0 (see discussion below). For these reasons, we consider the solutions from BAGPIPES to be more likely than the solutions from Prospector. Although we are unable to reject them on a formal basis, the Prospector fits would have radical implications for models of early galaxy evolution.

**Fig. 2:**
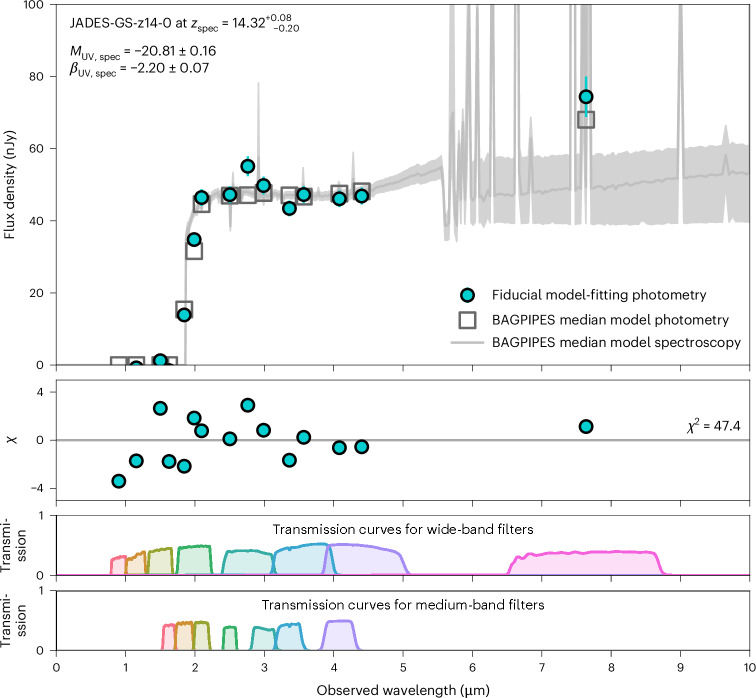
SED modelling for JADES-GS-z14-0. Upper panel: the measured spectral flux density and corresponding uncertainties are used to constrain the various SED models with BAGPIPES. As provided in Table 1, the mean and 1*σ* s.d. of the fiducial model-fitting photometry are the blue circles and error bars. The median of the SED models is the grey line and unfilled squares, while the 1*σ* confidence interval is the shaded region. Middle panel: the median model photometry is compared with the measured fluxes and uncertainties (*χ*), while the total *χ*^2^ value is reported on the right. Lower panels: the transmission curves for the various filters. These results suggest that the excess flux in F770W relative to F444W is from nebular emission-line contributions, while the underlying continuum is relatively flat at rest-frame optical wavelengths.

Figure 2 shows the measured photometry in all of the available NIRCam and MIRI filters, alongside the inferred SED determined with BAGPIPES. This fitting of the SED is self-consistently able to constrain the properties of the (1) stellar populations, (2) dust attenuation and (3) nebular gas. For simplicity, SEDs are modelled assuming a Kroupa stellar IMF^21^. Absorption from the IGM and attenuation from diffuse interstellar dust are included, along with nebular contributions from continuum and emission lines. To understand the effects of differing star-formation histories (SFHs), we ran models for (1) a parametric constant SFH, (2) a parametric delayed-tau SFH and (3) a non-parametric continuity SFH. Comparing the measured photometry and corresponding uncertainties (filled circles) with the median of the inferred photometry from the SED modelling (unfilled squares) demonstrates the success in the modelling. The 1*σ* confidence interval (shaded regions) suggests that the majority (that is, at least 50%, and up to 100%) of the excess flux in the F770W filter is from the nebular emission lines Hβ and [O iii]*λλ*4959, 5007, while the underlying continuum in the rest-frame optical regime is flat and consistent with the measured flux density in F444W.

**Fig. 3:**
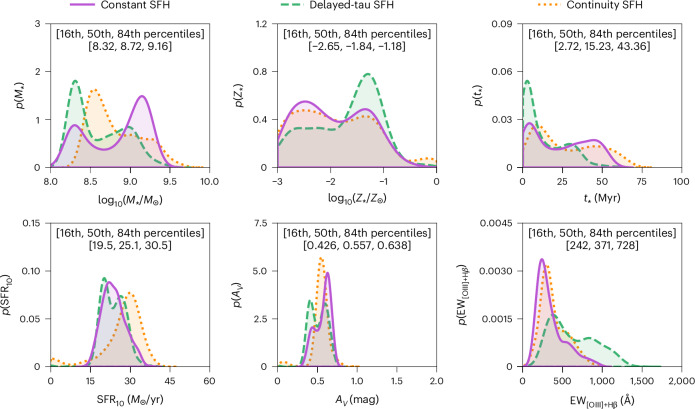
Stellar-population synthesis (SPS) modelling using the NIRCam and MIRI photometry. The measured fluxes and uncertainties are used to constrain the various SED models with BAGPIPES. Shown are the posterior distributions of *M*_★_, *Z*_★_, *t*_★_, SFR_10_, *A*_V_ and EW_[OIII]+Hβ_. We report the 16th, 50th and 84th percentiles after combining the posterior distributions from the various SED models. Three different SFHs are assumed: parametric constant SFH, parametric delayed-tau SFH and non-parametric continuity SFH.

**Extended Data Fig. 4:**
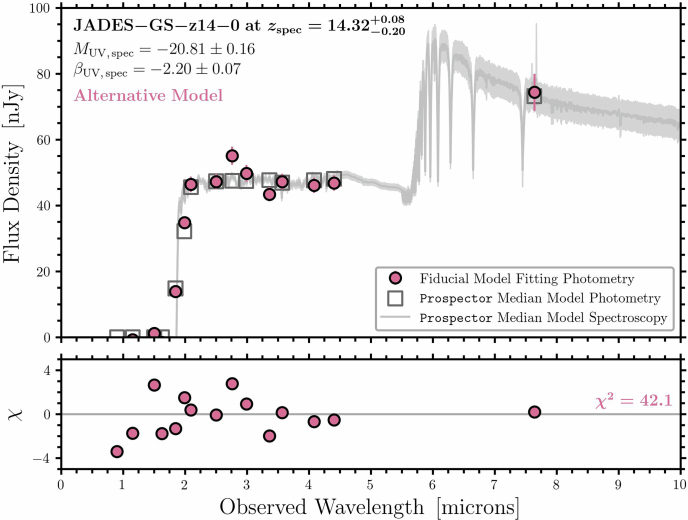
SED modeling for JADES-GS-z14-0. Similar to Fig. 2, the measured spectral flux density and corresponding uncertainties are used to constrain the various SED models with Prospector. As provided in Table 1, the mean and 1*σ* standard deviation of the fiducial model fitting photometry are the red circles and error bars. The median of the SED models is the gray line and unfilled squares, while the 1*σ* confidence interval is the shaded region. These results suggest that the excess flux in F770W relative to F444W is from stellar continuum rather than nebular emission line contributions, producing strong Balmer breaks in the models, which we find unlikely given the extreme inferred stellar masses and stellar ages.

Figure 3 and Extended Data Table 2 present the marginalized distributions for the BAGPIPES constraints on stellar mass (*M*_★_/*M*_⊙_, upper left), stellar metallicity (*Z*_★_/*Z*_⊙_, upper middle), *t*_★_ (Myr, upper right), star-formation rate averaged over the previous 10 million years (SFR_10_ (*M*_⊙_ yr^−1^), lower left), diffuse dust attenuation as measured in the V band (*A*_V_ (mag), lower middle) and rest-frame equivalent width of [O iii] + Hβ (EW_[OIII]+Hβ_ (Å), lower right). The equivalent width is measured for the combined rest-frame optical nebular emission lines Hβ and [O iii]*λλ*4959, 5007. Similarly, Extended Data Fig. 4 presents constraints on the joint posterior distributions and SFHs with BAGPIPES. The median derived *M*_★_ ≈ 10^8.7^ *M*_⊙_ (with a 1*σ* confidence interval of *M*_★_ ≈ 10^8.3^–10^9.2^ *M*_⊙_) is nearly one-tenth of the current value for the Milky Way. The median star-formation rate SFR ≈ 25 *M*_⊙_ yr^−1^ (with a 1*σ* confidence interval of SFR ≈ 20–31 *M*_⊙_ yr^−1^) is consistent with expectations based on the empirical star-forming main sequence derived at lower redshifts (*z* ≈ 8; for example, ref. ^22^). Taken together with the measured half-light radius, the star-formation rates imply star-formation rate surface densities *Σ*_SFR_ ≈ 64 *M*_⊙_ yr^−1^ pc^−^^2^ (with a 1*σ* confidence interval of *Σ*_SFR_ ≈ 50–78 *M*_⊙_ yr^−1^ pc^−^^2^), comparable to the most vigorous starbursts observed in the local Universe (see, for example, ref. ^23^).

The inferred stellar masses and star-formation rates are deduced from the emission produced by massive stars and an assumed ‘local’ IMF. However, it is likely that the formation of low-mass stars is strongly suppressed due to both the high temperature of the cosmic microwave background (*T* ≈ 60 K at *z* = 20; ref. ^24^) and the low metallicity of galaxies at *z* > 10 (refs. ^25,26^). Recent work has found that stellar masses can be reduced by factors of three (or more) by changing assumptions about the IMF, without affecting the resulting SED^27^, which our own analysis confirms.

Returning to the BAGPIPES constraints on physical properties, *t*_★_ ≈ 15 Myr (with a 1*σ* confidence interval of *t*_★_ ≈ 3–43 Myr) is consistent with values measured for some of the youngest galaxies observed at lower redshifts (*z* ≈ 8; see, for example, refs. ^28,29^). Similarly, median EW_[OIII]+Hβ_ ≈ 370 Å (with a 1*σ* confidence interval of EW_[OIII]+Hβ_ ≈ 240–730 Å), consistent with values measured for those same galaxies observed at lower redshifts (*z* ≈ 8; see, for example, refs. ^28,29^). The median *A*_V_ ≈ 0.56 AB mag (as measured in the V band, with a 1*σ* confidence interval of *A*_V_ ≈ 0.43–0.64 AB mag) suggests a small amount of attenuation at rest-frame optical wavelengths. A detailed discussion and interpretation of the dust content for JADES-GS-z14-0 is presented in ref. ^11^. Finally, the median *Z*_★_ ≈ 0.014 *Z*_⊙_ (with a 1*σ* confidence interval of *Z*_★_ ≈ 0.002–0.066 *Z*_⊙_) is largely unconstrained but consistent with metallicities that are less than 10% of the solar value. These results agree with those determined in ref. ^11^, which derives a stellar metallicity from SED modelling with BEAGLE^30^. Assuming that the stellar and gas-phase metallicities are the same, they find ${\log }_{10}\left({\rm{O}}/{\rm{H}}\right)+12=7.{2}_{-0.4}^{+0.7}$. Our own SED fitting finds an equivalent gas-phase metallicity of ${\log }_{10}\left({\rm{O}}/{\rm{H}}\right)+12=6.{9}_{-0.8}^{+0.7}$.

**Extended Data Fig. 5:**
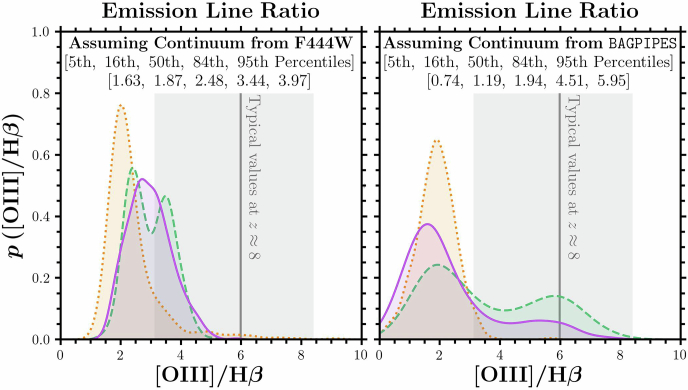
[OIII]/H*β* emission line ratio predictions for JADES-GS-z14-0. The physical properties inferred from SED modeling with BAGPIPES are used to predict the emission line ratio [OIII]/H*β*. This requires an assumption about the underlying continuum at rest-frame optical wavelengths. The left panel assumes the continuum is flat and consistent with the measured flux density in F444W, while the right panel assumes the posterior distribution of continuum levels from BAGPIPES. Various SED models with different SFH assumptions (constant in purple, delayed-tau in green, and continuity in orange) are implemented. We report the 5th, 16th, 50th, 84th, and 95th percentiles after combining the posterior distributions from the various SED models. For comparison, we include measurements for a sample of galaxies at z ≈ 8 with H*β* line flux measurements. The median of this sample is the gray lines, while the 1*σ* confidence interval is the shaded regions.

The gas-phase metallicity encodes valuable information about the baryonic processes shaping the formation and evolution of galaxies. It has been found to be correlated with the emission-line ratio [O iii]/Hβ, which compares the strengths of the collisionally excited [O iii]*λλ*4959, 5007 lines with the Balmer recombination Hβ line. We can place loose constraints on this emission-line ratio by combining the stellar-population properties (and the predicted rest-frame optical continuum) with the measured excess flux in F770W relative to F444W. We proceed analogously to the fundamental relations derived in ref. ^31^ and compare inferred star-formation rates with observed Hβ line luminosities. The relationship between these two quantities is derived for a sample of galaxies at *z* ≈ 8 with Hβ line flux measurements from NIRSpec/PRISM observations, which are from JWST Advanced Deep Extragalactic Survey (JADES) Data Release 3 (DR3)^32^. Combining our derived calibration with the inferred star-formation rates from BAGPIPES yields an Hβ line flux ${F}_{{\rm{H}}\upbeta }=7.{9}_{-1.8}^{+1.7}\times 1{0}^{-19}\,{\rm{ergs}}\,{\rm{s}}^{-1}\,{{\rm{cm}}}^{-2}$. Comparing this derived quantity with two assumptions about the underlying continuum at rest-frame optical wavelengths yields $[{\rm{O}}\,{\text{III}}]/{\rm{H}}\upbeta =2.{5}_{-0.6}^{+0.9}$ and $1.{9}_{-0.7}^{+2.6}$ (Extended Data Fig. 5). For comparison, the typical value is [O iii]/Hβ ≈ 6 for the aforementioned sample of galaxies at *z* ≈ 8 from JADES DR3^32^. These results imply that the measured excess flux at 7.7 μm includes a substantial contribution from [O iii]*λλ*4959, 5007, but with a gas-phase metallicity that is smaller than typical values at *z* ≈ 8 (see discussion in Methods).

The detection of JADES-GS-z14-0 at *z* > 14 by MIRI demonstrates its power in understanding the properties of the earliest galaxies. The most plausible solution for the flux observed at 7.7 μm with MIRI suggests substantial contributions from the nebular emission lines [O iii]*λλ*4959, 5007, which indicates metal enrichment for this galaxy. An alternative model is possible, but less likely, since it suggests an extreme stellar mass and a strong Balmer break from evolved stars. Deep spectroscopic follow-up observations with MIRI's low-resolution spectrometer are required to disentangle these interpretations by directly measuring the contributions to this flux from the nebular emission lines Hβ and [O iii]*λλ*4959, 5007. Such observations would also include Hα, producing a direct measurement of the star-formation rate. Given the size, luminosity and redshift of JADES-GS-z14-0, these measurements would build on a truly unique opportunity to study galaxy formation when the Universe was less than 300 Myr old.

## Methods

Throughout this work, we report wavelengths in air and adopt the standard flat *Λ*CDM cosmology from Planck18 with *H*_0_ = 67.4 km s^−1^ Mpc^−1^ and *Ω*_m_ = 0.315 (ref. ^33^). All magnitudes are in the AB system^34^. Uncertainties are quoted as 68% (1*σ*) confidence intervals, unless otherwise stated.

### Observations

The observations used in this work consist of infrared imaging with NIRCam and MIRI in the Great Observatories Origins Deep Survey South (GOODS-S)^35^ field, near the Hubble Ultra Deep Field^36^ and the JADES Origins Field^37^. The NIRCam data were primarily observed as part of JADES^38^, but also as part of the First Reionization Epoch Spectroscopic Complete Survey^39^. The MIRI data were observed as part of JADES.

JADES-GS-z14-0 was observed with NIRCam in four separate programmes, which we separate into three categories on the basis of observing time. Ultradeep observations were conducted with programme IDs 1210 (N. Lützgendorf) and 3215 (D.J.E.) across an area of roughly 10 arcmin^2^ using 14 photometric filters, including seven wide bands (F090W, F115W, F150W, F200W, F277W, F356W and F444W) and seven medium bands (F162M, F182M, F210M, F250M, F300M, F335M and F410M). These were observed on 20–24 October 2022 (for programme ID 1210) and 16–24 October 2023 (for programme ID 3215), with a range in integration times of 39–73 h for each of the wide bands and 55–165 h for each of the medium bands, reaching 5*σ* depths of 1.9–3.0 nJy and 1.6–2.6 nJy, respectively. Medium-depth observations were conducted with programme ID 1180 (D.J.E.) across an area of roughly 40 arcmin^2^ using eight filters, including the same seven wide bands as programme ID 1210 plus one medium band (F410M). These were observed on 29 September–5 October 2022 and 28 September–3 October 2023, with a range in integration times of 6–8 h for each of the filters, reaching 5*σ* depths of 4.1–7.3 nJy. Shallow observations were conducted with programme ID 1895 (P. Oesch) across an area of roughly 60 arcmin^2^ using three filters (F182M, F210M and F444W). These were observed on 13–18 November 2022, with a range in integration times of 0.25–1.25 h, reaching 5*σ* depths of 10–12 nJy. Depths are estimated using 0.2-arcsec-radius circular apertures assuming point-source morphologies. The aforementioned NIRCam observations have been presented and discussed extensively in the literature (for example, ^4,5,37–40^).

JADES-GS-z14-0 was also observed with MIRI as a coordinated parallel to NIRCam with programme ID 1180 (D.J.E.). The MIRI imaging includes four pointings near the JADES Origins Field at a position angle of 300°, which together produce an ultradeep contiguous mosaic of roughly 8.8 arcmin^2^. In all four of the pointings, we conducted two separate nine-point dithers of 1,361 second individual exposures with MIRI for five different NIRCam filter pairs (2 × 9 × 5 total exposures), for a total exposure time of roughly 61.3 ks with MIRI for each of the two dithers. We conducted two additional four-point dithers of 1,361 second individual exposures with MIRI for three different NIRCam filter pairs (2 × 4 × 3 total exposures), for a total exposure time of roughly 16.3 ks for each of the two dithers. The readout mode SLOWR1 was utilized with 57 groups to minimize data volume. These data were obtained on 29 September–5 October 2022 and 28 September–3 October 2023, with a typical on-source integration time of roughly 43.1 h (155.2 ks), reaching a 5*σ* depth of 21 nJy (28.1 AB mag) using 0.4-arcsec-radius circular apertures and assuming point-source morphologies as before. At the location of JADES-GS-z14-0, which falls near the edge of the MIRI imaging, the typical on-source integration time is roughly 23.8 h (85.7 ks) which corresponds to a 5*σ* depth of 28 nJy (27.8 AB mag). These MIRI observations have been presented and discussed previously at half depth in the literature (for example, ref. ^41^). This work is a presentation and discussion of the full-depth MIRI observations, which are some of the deepest images ever taken in the mid-infrared, alongside the ultradeep MIRI Deep Imaging Survey results in the Hubble Ultra Deep Field at 5.6 μm (refs. ^42,43^). The observations presented here will be described in more detail in a forthcoming paper from the JADES collaboration (S.A. et al., manuscript in preparation).

### Image reduction

A detailed description of the reduction, mosaicing, source detection and photometric measurements for the NIRCam data is provided in the first JADES data release in GOODS-S^40^ and in ref. ^11^. Similarly, a detailed description of the reduction, mosaicing, source detection and photometric measurements for the MIRI data will be provided in an upcoming JADES data release in GOODS-S (S.A. et al., in preparation), but has already been partially introduced^41,44,45^. We briefly summarize the main steps of the reduction and mosaicing process for the MIRI data. Raw images are processed with the JWST Calibration Pipeline (v.1.12.5)^46^ using the Calibration Reference Data System pipeline mapping 1188, similar to the procedure with NIRCam data. We run stage 1 of the JWST Calibration Pipeline using all of the default parameters, plus a correction for cosmic ray showers. Stage 2 is run using all of the default parameters, plus an additional custom subtraction of the background using our own super sky backgrounds. Following this step, we perform an additional astrometric correction. Finally, stage 3 is run using all of the default parameters, but without any further alignment or matching. The final image mosaic is registered to the Gaia DR3 frame^47^ and resampled onto the same world coordinate system as the NIRCam image mosaics, but with a 0.060 arcsec per pixel grid.

### Detection and photometry

To interpret the ultradeep MIRI observations in the context of the ultradeep NIRCam observations, we measure the flux density in MIRI/F770W relative to the flux density in one of the long-wavelength NIRCam filters (that is, F444W). The MIRI detection is of modest *S*/*N* ≈ 13 with respect to all of the NIRCam detections (*S*/*N* ≈ 25–100). Furthermore, the MIRI/F770W diffraction-limited PSF (FWHM of 0.269 arcsec) is larger than that of NIRCam/F444W (FWHM of 0.145 arcsec) by nearly a factor of two. JADES-GS-z14-0 and the neighbouring foreground galaxy to the east, NIRCam ID 183349, have similar measured flux densities in F444W and are both morphologically extended (FWHMs of the deconvolved light profiles are both roughly 0.16 arcsec)^11^. These qualities make the separation (or deblending) of JADES-GS-z14-0 from 183349 challenging, but essential for physical interpretation of the MIRI observations.

#### Detailed model-fitting photometry

Our primary approach for measuring photometry uses detailed model fitting in the vicinity of JADES-GS-z14-0 with the Bayesian fitting code ForcePho (B.D.J. et al., manuscript in preparation). The motivation for performing these more complicated photometric measurements is the complexity of the region surrounding JADES-GS-z14-0, with multiple bright foreground galaxies within a radius of a few arcseconds. It is important to properly disentangle the relative flux contributions from these galaxies. Furthermore, this region of the sky is located near the edge of the MIRI exposures, which may affect measurements of the photometric uncertainties. We briefly summarize here the main steps in measuring the detailed model-fitting photometry. Since the galaxies are better resolved with NIRCam, we determine their morphological properties (that is, half-light radius, Sérsic index, axis ratio, position angle and total flux for each NIRCam filter) by fitting the NIRCam data, and then use the results to constrain the MIRI photometry.

The first step for the NIRCam data is to construct 2.5 arcsec × 2.5 arcsec cutouts from all of the available exposures, with each of these cutouts centred on the source location of JADES-GS-z14-0 from the original photometric catalogue. Cutouts are performed on exposures after reduction but before mosaicing. All of the detected sources within these cutouts are simultaneously modelled to determine the relative flux contributions. These models assume an intrinsic Sérsic profile that is identical for all the different NIRCam filters^48^. We convolve these intrinsic light profiles with Gaussian mixture approximations to the PSF in each of the NIRCam exposures to produce observed light profiles. We utilize the results of this detailed model-fitting approach by taking the mean and s.d. of the resulting total flux posterior distributions, which are sampled with Markov chain Monte Carlo techniques and account for the covariance between parameters.

JADES-GS-z14-0 and 183349 are significantly detected (*S*/*N* > 5) in the vast majority of the individual NIRCam exposures, but they are only marginally detected (*S*/*N* = 2–3) in most of the individual MIRI exposures. This necessitates different detailed model-fitting photometric procedures for NIRCam and MIRI. Extended Data Figure 1 illustrates the photometric modelling process used to measure the flux density in F770W. The first step is once again to construct 2.5 arcsec × 2.5 arcsec cutouts, now with all of the available MIRI exposures. All of the detected sources within these cutouts are simultaneously modelled by adopting the inferred morphological properties from the ForcePho fitting to the NIRCam data (for example, half-light radius, Sérsic index, axis ratio and position angle). For JADES-GS-z14-0, the inferred morphological parameters are half-light radius *r*_1/2_ = 0.0788 ± 0.0006 arcsec, Sérsic index *n* = 0.877 ± 0.027, axis ratio *b*/*a* = 0.425 ± 0.008 and position angle PA = 1.427 ± 0.008 rad. For 183349, the inferred parameters are *r*_1/2_ = 0.0872 ± 0.0006 arcsec, *n* = 0.815 ± 0.020, *b*/*a* = 0.378 ± 0.006 and PA = 4.171 ± 0.003 rad. The adopted Sérsic profile is used to forward-model the total flux in the F770W filter with GALFIT^14,15^. We adopt the mean and s.d. of the resulting total flux posterior distributions as fiducial for MIRI. For JADES-GS-z14-0, we measure *f*_F444W_ = 46.9 ± 0.6 nJy and *f*_F770W_ = 74.4 ± 5.6 nJy, corresponding to Δ*f* = 27.5 ± 5.6 nJy in F770W with respect to F444W. We report these measurements in Table 1. The uncertainties are additionally validated using two different methodologies, producing consistent results within 10% of one another: (1) bootstrapping individual exposures and (2) measuring photometry in regions of the sky that are observed to be empty in the much deeper NIRCam images. For 183349, we measure *f*_F444W_ = 48.9 ± 0.6 nJy and *f*_F770W_ = 46.3 ± 4.6 nJy.

The model-fitting approach assumes that JADES-GS-z14-0 and 183349 are well described by single Sérsic light profiles. To explore the impact of this assumption, we additionally adopt a non-parametric approach. We construct galaxy templates by deconvolving the NIRCam images in the F150W and F444W filters, where the F150W image corresponds to 183349 alone, while the F444W image corresponds to both JADES-GS-z14-0 and 183349. We conduct the deconvolution using the Wiener–Hunt method and Gaussian mixture approximations for the PSFs to mitigate noise amplification at high frequencies. We then convolve the templates into the MIRI/F770W band and fit the amplitudes of the two templates simultaneously, where we measure on individual exposures and analytically derive the best estimated values and covariances as before. We assign zero weight to regions below a significance of 3*σ* to prevent overfitting. Finally, we convert the template amplitude to galaxy fluxes according to the ForcePho photometry results in the NIRCam F150W and F444W bands. This method yields *f*_F770W_ = 69.3 ± 6.1 nJy for JADES-GS-z14-0, which is consistent with the parametric model-fitting photometry. Moreover, we apply this non-parametric approach to measure fluxes across all of the NIRCam filters, obtaining results consistent with the reported photometry in Table 1. For 183349, this method yields *f*_F770W_ = 47.8 ± 4.0 nJy.

#### PSF photometry

Our primary measurements are based on the model-fitting approach described above. Alternatively, we could treat both galaxies as point sources, since their measured half-light angular sizes are notably smaller than the F770W PSF. Therefore, as a check on the model-fitting result, we also perform PSF photometry. To measure the observed flux density in F770W relative to F444W, we first convolve the F444W mosaic to the PSF of the F770W filter, which involves convolving the F444W mosaic with a kernel of the difference between the F444W and F770W PSFs. The adopted MIRI PSF is empirically measured from the final image mosaic and accounts for the ‘cruciform’ detector artefact^49^. We simultaneously fit for the flux densities of JADES-GS-z14-0 and 183349 in both the convolved F444W and unconvolved F770W image mosaics. Extended Data Figure 2 illustrates the photometric modelling process used to measure the flux density in F770W. The residual images (shown in the right-hand panel) illustrate the validity of the point-source assumption and the success in the photometric modelling. For JADES-GS-z14-0, we measure *f*_F444W_ = 46.4 ± 1.2 nJy and *f*_F770W_ = 64.7 ± 6.1 nJy, corresponding to Δ*f* = 18.3 ± 6.2 nJy in F770W with respect to F444W. The uncertainty on the F770W flux density is found by injecting fake point sources into the final image mosaic, extracting them with procedures identical to those used for JADES-GS-z14-0, and then calculating the sigma-clipped s.d. of the difference between the recovered and injected flux densities. These results are consistent with those obtained using both the parametric and non-parametric model-fitting approaches. The measured F770W flux density is slightly smaller for the PSF photometry, consistent with a small amount (roughly 10%) of galaxy light falling far enough outside the image cores that it is not included in this photometric approach.

#### Conclusion

To summarize, we obtain consistent results for the NIRCam and MIRI photometry of JADES-GS-z14-0 using three different methods. The PSF fitting approach provides a simple baseline that is free from complex modelling assumptions. The model-fitting approach adopts both parametric and non-parametric galaxy models while measuring photometry from the individual exposures. This method more accurately accounts for the extended morphologies of JADES-GS-z14-0 and 183349, which improves measurements of the diffuse galaxy flux. This extended flux is the source of the larger F770W flux densities that are measured with the model-fitting approach when compared with the simpler PSF fitting approach.

### SPS modelling

The combination of ultradeep NIRCam, NIRSpec and MIRI observations provides an unprecedented opportunity to study the physical properties of JADES-GS-z14-0. We utilize the Bayesian SED fitting code BAGPIPES^16^ to self-consistently model the properties of the stellar populations, dust attenuation and nebular gas. We choose to sample the posterior distributions of these derived properties with the importance nested sampling code nautilus^50^, assuming an effective sample size of 10^4^. Fits are performed on the fiducial model-fitting photometry after imposing an error floor of 5%. Such an error floor is imposed since the quoted photometric uncertainties are probably underestimates of the true errors. This is because we do not account for systematic uncertainties related to, for example, imperfect photometric calibration, background subtraction and/or parametric assumptions about the intrinsic light profiles (where we assume a single Sérsic profile for each source). We briefly summarize here the various components of the assumed physical model.

Stellar populations are derived using predefined SPS models^30^, which are the 2016 updated version of previous models^51^, and are determined for a grid of simple stellar-population models with various ages and metallicities. We adopt the stellar library from the Medium-Resolution Isaac Newton Telescope Library of Empirical Spectra^52^, in addition to the stellar evolutionary tracks and isochrones from the Padova and Trieste Stellar Evolution Code^53^. We assume a Kroupa IMF^21^ with a lower bound of 0.08 *M*_⊙_ and an upper bound of 120 *M*_⊙_. Absorption from the IGM is modelled after ref. ^54^, which is an updated version of the model from ref. ^55^. Dust attenuation is modelled after ref. ^56^ with one free parameter: *A*_V_, assuming a uniform prior with min = 0.0 and max = 2.0. Nebular emission (from both emission lines and continuum) is modelled after ref. ^57^ using the photoionization code Cloudy^58^ with one free parameter: the ionization parameter (log_10_(*U*), assuming a uniform prior with min = −4.0 and max = −2.0). The gas-phase metallicity is fixed to the value of the stellar metallicity.

To explore the impact of assuming different SFHs on the results, we model the SEDs with two parametric SFHs (constant model and delayed-tau model) and one non-parametric SFH (continuity model). Each of the assumed SFHs has at least two free parameters: the total stellar mass formed (log_10_(*M*_★_/*M*_☉_), assuming a uniform prior with min = 6.0 and max = 12.0) and the stellar metallicity (log10(*Z*_★_/*Z*_☉_), assuming a uniform prior with min = −3.0 and max = 0.0). The constant-SFH model has one additional free parameter: the galaxy age (*t* (Myr), assuming a uniform prior with min = 1.0 and max = *t*_umiv_ (Myr), where *t*_univ_ is the age of the Universe measured with respect to the formation redshift *z*_form_ = 20). The delayed-tau SFH model has two additional free parameters: *t* (Myr), assuming a uniform prior with min = 1.0 and max = *t*_univ_ (Myr), and the *e*-folding time for the delayed-tau component (*τ* (Gyr), assuming a log-uniform prior with min = 0.001 and max = 30.0). Finally, the continuity SFH model has four additional free parameters, corresponding to the logarithm of the ratio of the star-formation rates in the five adjacent time-bins (*R*_SFR_, assuming Student’s *t*-distribution prior with *μ* = 0.0, *σ* = 0.3). These time-bins are spaced at lookback times of 0–3, 3–10, 10–30, 30–100 and 100–*t*_univ_ Myr, which assumes that the SFH starts at *z*_form_ = 20. These physical models have between five and eight free parameters, which should be compared with the 11 photometric detections that we have for JADES-GS-z14-0 (F182M, F200W, F210M, F250M, F277W, F300M, F335M, F356W, F410M, F444W and F770W).

Figure 3 presents the marginalized distributions for the BAGPIPES constraints on *M*_★_, *Z*_★_, *t*_★_, SFR_10_, *A*_V_ and EW_[OIII]+Hβ_. Results from each of the assumed SFHs are shown: the parametric constant-SFH model is represented by the purple solid lines, the parametric delayed-tau SFH model by the green dashed lines and the non-parametric continuity SFH model by the orange dotted lines. Throughout, we quote median derived properties and 1*σ* confidence intervals by concatenating the results from each of these SFHs. These measurements are reported in Extended Data Table 1, alongside measurements of the star-formation rate surface density (${\Sigma }_{{{\rm{SFR}}}_{10}}$), specific star-formation rate (sSFR_10_) and log_10_(*U*).

Similar to Fig. 3, Extended Data Fig. 4 shows the joint posterior distributions for some of the inferred physical parameters in the lower left, alongside the derived SFHs in the upper right. Results from each of the assumed SFHs are provided: the parametric constant-SFH model is shown in purple, the parametric delayed-tau SFH model in green and the non-parametric continuity SFH model in orange. The inferred stellar masses have two peaks in their posterior distributions, one at low *M*_★_ ≈ 10^8.5^ *M*_⊙_ and one at high *M*_★_ ≈ 10^9.0^ *M*_⊙_. The low-mass solutions suggest less stellar continuum at rest-frame optical wavelengths, and therefore larger equivalent widths of the rest-frame optical nebular emission lines Hβ and [O iii]*λλ*4959, 5007. The opposite is true for the high-mass solutions, where the equivalent widths are smaller due to more stellar continuum in the rest-frame optical wavelengths. The delayed-tau and continuity SFHs prefer the low-mass solution, while the constant SFH finds equal weight for the two solutions. For all of the models, stellar mass is degenerate with stellar age, where the low-mass solution corresponds to younger stellar populations (with mass-weighted ages of a few million years), while the high-mass solution corresponds to older stellar populations (with ages of a few tens of millions of years). Some of the models suggest more extended periods of star formation out to lookback times of up to 100 Myr, which would be enough time to enrich this galaxy via type II supernovae. Despite these differences, the rest of the inferred physical parameters are similar for the two stellar-mass and stellar-age solutions. All of the inferred physical parameters are fairly well constrained by the existing observations, except for the stellar metallicity (and therefore also the gas-phase metallicity). The results of our SED modelling agree within the quoted uncertainties with the SED modelling of ref. ^11^, which includes JWST/NIRSpec data and adopts various physical models with BEAGLE^30^.

To explore the impact of adopting different fitting codes, we additionally utilize the Bayesian SED fitting code Prospector (v.1.2.0)^17^ to self-consistently model the properties of the stellar populations, dust attenuation and nebular gas. We choose to sample the posterior distributions of these derived properties with the dynamic nested sampling code dynesty(v.1.2.3)^59^, assuming an effective sample size of 10^4^. Fits are performed on the model-fitting photometry after imposing an error floor of 5%. The assumed physical model closely follows that of the BAGPIPES modelling, and we briefly summarize here the various components of this model.

Stellar populations are derived with the Flexible Stellar Population Synthesis code (FSPS)^60,61^, which is accessed through the python-fsps bindings^62^. Stellar evolution is computed by the Modules for Experiments in Stellar Astrophysics package (MESA)^63–66^, while using the synthetic models from MESA Isochrones and Stellar Tracks^67,68^. The stellar libraries, in addition to the stellar evolutionary tracks and isochrones, are the primary differences between the physical models that we assumed for BAGPIPES and Prospector. Thus, we attribute any differences in the derived physical properties to these differences in the stellar libraries, stellar evolutionary tracks and isochrones. We assume a Kroupa IMF^21^ with a lower bound of 0.08 *M*_⊙_ and an upper bound of 120 *M*_⊙_. Absorption from the IGM is modelled after ref. ^55^. Dust attenuation is modelled after ref. ^56^ with one free parameter: *A*_V_, assuming a uniform prior with min = 0.0 and max = 2.0. Nebular emission (from both emission lines and continuum) is modelled after ref. ^57^ using the photoionization code Cloudy^58^ with one free parameter: log_10_(*U*), assuming a uniform prior with min = −4.0 and max = −1.0). The gas-phase metallicity is fixed to the value of the stellar metallicity.

Finally, we assume the continuity model for the SFH. This is the only SFH that we assume for Prospector, since the two parametric SFHs (constant model and delayed-tau model) are unable to reproduce the observed photometry without invoking additional free parameters (for example, the escape fraction, or the shape of the diffuse dust attenuation curve). The inclusion of additional free parameters, such as the escape fraction, reduces the inferred stellar masses by roughly 0.5 dex without affecting any of the other inferred physical parameters. This SFH has two free parameters, corresponding to the total stellar mass formed (log_10_(*M*_★_/*M*_☉_)), assuming a uniform prior with min = 6.0 and max = 12.0), and the stellar metallicity (log_10_(*Z*_★_/*Z*_☉_), assuming a uniform prior with min = −3.0 and max = 0.0). It also has four additional free parameters, corresponding to the logarithm of *R*_SFR_, assuming Student’s *t*-distribution prior with *μ* = 0.0, *σ* = 0.3. These time-bins are spaced at lookback times of 0–3, 3–10, 10–30, 30–100 and 100–*t*_univ_ Myr, which assumes that the SFH starts at *z*_form_ = 20. This physical model has eight free parameters, which should be compared with the 11 photometric detections that we have for JADES-GS-z14-0.

Similar to Fig. 2, Extended Data Fig. 3 shows the SED modelling for JADES-GS-z14-0 with Prospector. In the upper panel, the fiducial model-fitting photometry is used to constrain the various SED models. The median of these models is the grey line and unfilled squares, while the 1*σ* confidence interval is the shaded region. Extended Data Table 2 reports the 16th, 50th and 84th percentiles for the inferred physical properties. In the lower panel, the median model photometry is compared with the measured fluxes and uncertainties (*χ*), while the total *χ*^2^ value is reported on the right. Prospector suggests that the excess flux in F770W relative to F444W is from stellar continuum rather than nebular emission-line contributions, producing strong Balmer breaks in the models, which we find unlikely for the following reasons.

1. Prospector predicts stellar masses of *M*_★_ ≈ 10^9.4^ *M*_⊙_ that are nearly an order of magnitude larger than for the BAGPIPES models (*M*_★_ ≈ 10^8.7^ *M*_⊙_). Reference ^5^ demonstrated that halos with virial masses of *M*_★_ ≈ 10^9.8−9.9^ *M*_⊙_ have abundances comparable to those of nine galaxy candidates at *z* = 12–15, including JADES-GS-z14-0. This implies that the Prospector-predicted stellar mass is only three times smaller than the halo mass predicted through abundance matching, which is smaller than the predicted stellar-to-halo-mass relation at *z* ≈ 14 (see, for example, ref. ^69^).
2. Theoretical predictions from the First Light and Reionization Epoch Simulations (FLARES)^19^ suggest that galaxies with *M*_★_ ≳ 10^9.0^ *M*_⊙_ only exist at *z* < 14, while galaxies with *M*_★_ ≳ 10^9.5^ *M*_⊙_ only exist at *z* < 13. Additionally, theoretical predictions from the IllustrisTNG and THESAN projects^70^ suggest that galaxies with *M*_★_ ≳ 10^9.0^ *M*_⊙_ only exist at *z* < 12. Thus, cosmological galaxy formation simulations do not predict any galaxies at *z* ≈ 14 with stellar masses that are comparable to those predicted by the Prospector models.
3. The issues related to the extreme stellar masses predicted by Prospector are exacerbated by the extreme stellar ages (*t*_★_ ≈ 80–100 Myr, corresponding to formation redshifts of *z* ≈ 18–20). Given that cosmological simulations are unable to produce galaxies at *z* ≈ 14 with stellar masses that are comparable to those predicted by the Prospector models, they certainly cannot produce those same galaxies at *z* ≈ 18–20. On the other hand, the stellar masses, stellar ages, specific star-formation rates and diffuse dust attenuations predicted by the BAGPIPES models are consistent with theoretical predictions from FLARES^19^.

If they were correct, the physical properties predicted by the Prospector models would have radical implications for models of galaxy evolution in the early Universe, since cosmological simulations do not predict any galaxies at the redshift frontier with these extreme inferred stellar masses and stellar ages. For the reasons outlined above, we consider the solutions from BAGPIPES to be more plausible than the solutions from Prospector, but we are unable to reject either of these solutions on a formal basis. As a reminder, we attribute any differences in the predicted physical properties between BAGPIPES and Prospector to differences in the assumed stellar libraries, in addition to the stellar evolutionary tracks and isochrones.

Bayesian SED fitting requires numerous assumptions to interpret observations and infer physical properties for galaxies. We have explored the impact of adopting different SFHs and fitting codes, where the former had very little impact on the derived stellar-population properties while the latter had substantial impact. Another assumption that can have substantial impact on the derived stellar-population properties is the IMF. In particular, it is likely that the formation of low-mass stars (that is, those with *M*_★_ < 1–3 *M*_⊙_) is strongly suppressed at high redshifts. This is caused by the high temperature of the cosmic microwave background^24^ and the low metallicity of galaxies at *z* > 10 (refs. ^25,26^). To explore the impact of these physical processes, we utilize the Bayesian SED fitting code Prospector, since BAGPIPES does not allow changes to the assumed IMF. With regard to top-heavy IMFs, Prospector provides the more demanding test because it fits the stellar continuum with older, low-mass stars whose formation is likely to be suppressed. For simplicity, we will assume the same Kroupa IMF^21^ as before, but vary the lower and upper bounds of the mass range. Increasing the lower bound from 0.08 *M*_⊙_ to 1 *M*_⊙_ (3 *M*_⊙_) decreases the inferred stellar masses by roughly 0.3 dex (0.4 dex), while increasing the upper bound from 120 *M*_⊙_ to 300 *M*_⊙_ has no effect on the inferred stellar masses. These results suggest that the reported stellar masses based on a local IMF can be overestimated by up to a factor of three without affecting the resulting SED. Recent work has found similar conclusions by changing assumptions about the IMF^27^.

### Nebular emission-line flux predictions

The aforementioned stellar-population and dust properties provide predictions for the strength of the Balmer hydrogen recombination lines Hβ and Hα. With an estimate of the strength of Hβ, it is possible to determine the remaining excess flux in F770W relative to F444W, which can be attributed to the metallic collisionally excited lines [O iii]*λλ*4959, 5007. These predictions require a series of steps and assumptions to convert the inferred star-formation rates into emission-line flux predictions then broadband flux predictions.

The first step in this process involves converting the BAGPIPES-derived star-formation rate into an Hβ line luminosity. To accomplish this, we proceed analogously to the fundamental relations derived in ref. ^31^. We compare BAGPIPES-derived star-formation rates with observed Hβ line luminosities for a sample of *N* = 27 galaxies at *z* ≈ 8 with Hβ line flux measurements from NIRSpec/PRISM. These measurements, along with Kron photometry convolved to the F444W PSF, are from JADES DR3^32^. We select all sources in GOODS-S with NIRCam coverage and *S*/*N* > 3 for the Hβ line flux measurements. The measured photometry is used to constrain the various SED models with BAGPIPES, with procedures identical to those used for JADES-GS-z14-0, assuming the same three SFHs and concatenating the results from each. This produces a distribution of calibrations for each of the galaxies, which we can combine to obtain the full distribution of calibrations for the entire sample. They produce the following calibration:

1 $${C}_{{\rm{H}}\upbeta }=\frac{{L}_{{\rm{H}}\upbeta }\,({\rm{ergs}}\,{\rm{s}}^{-1})}{{{\rm{SFR}}}_{10}\,({M}_{\odot }\,{\rm{yr}}^{-1})}=9.1\pm 5.6\times 1{0}^{40}.$$

The reported calibration and corresponding uncertainty arise from taking the sigma-clipped mean and s.d. of the full distribution of calibrations. The quoted uncertainty reflects the scatter around the average relation for individual galaxies. The dominant term in this uncertainty originates from the derived star-formation rates. Since the derived star-formation rate uncertainties are propagated throughout, we do not propagate the calibration uncertainty for the remaining calculations. The reported calibration results in a predicted Hβ line luminosity ${L}_{{\rm{H}}\upbeta }\approx 23{0}_{-150}^{+150}\times 1{0}^{40}\,{\rm{ergs}}\,{\rm{s}}^{-1}$.

The second step in this process involves converting the Hβ line luminosity into an Hβ line flux. This requires a luminosity distance, *d*_*L*_ = 4.803 × 10^29^ cm at *z* = 14.32, which is dependent on the assumed cosmology. This results in a predicted Hβ line flux ${F}_{{\rm{H}}\upbeta }\approx 7.{9}_{-1.8}^{+1.7}\times 1{0}^{-19}\,{\rm{ergs}}\,{\rm{s}}^{-1}\,{{\rm{cm}}}^{-2}$.

The third step in this process involves converting the Hβ line flux into the equivalent signal in the F770W band. This requires the effective bandwidth of the F770W filter, *W*_ν,F770W_ = 1.00 × 10^13^ Hz, as measured in frequency space and reported in the JWST User Documentation for the MIRI Filters and Dispersers. This results in a predicted Hβ F770W flux density ${f}_{{\rm{H}}\upbeta ,{\rm{F}}770{\rm{W}}}\approx 7.{9}_{-1.8}^{+1.7}\,{\rm{nJy}}$.

The final step in this process involves comparing the Hβ F770W flux density with the total nebular emission-line contribution to F770W. These nebular contributions are equal to the F770W flux density minus the total continuum contribution to F770W. For the continuum contribution to F770W, we first assume that the continuum is flat at rest-frame optical wavelengths and consistent with the measured flux density in F444W. We measure Δ*f* = 27.5 ± 5.6 nJy in F770W relative to F444W, which results in a predicted line ratio $[{\rm{O}}\,{\text{III}}]/{\rm{H}}\upbeta \approx 2.{5}_{-0.6}^{+0.9}$, as illustrated in the left-hand panel of Extended Data Fig. 5. The quoted uncertainties on the line ratio include uncertainties on the star-formation rates, but not on the rest-frame optical continuum. To account for uncertainties in the rest-frame optical continuum, we further assume the posterior distribution of continuum levels from BAGPIPES. This results in a predicted line ratio $[{\rm{O}}\,{\textsc{III}}]/{\rm{H}}\upbeta \approx 1.{9}_{-0.7}^{+2.6}$, as illustrated in the right-hand panel of Extended Data Fig. 5. Regardless of the assumption about the rest-frame optical continuum, we find that the excess flux at 7.7 μm includes a substantial contribution from the [O iii]*λλ*4959, 5007 lines. This line ratio is smaller than typical values observed for galaxies at *z* ≈ 8, as shown in Extended Data Fig. 5 with individual galaxies that have Hβ line flux measurements from JADES DR3^32^.

The line ratio [O iii]/Hβ is found to be correlated but degenerate with the gas-phase metallicity, with an additional, secondary dependence on the ionization and excitation states of the nebular gas. The turnover in the relation between [O iii]/Hβ and gas-phase metallicity occurs for *Z*_★_ ≈ 25% *Z*_⊙_ (see, for example, ref. ^71^), where lower values correspond to lower stellar masses, and higher values correspond to higher stellar masses. Given the inferred stellar masses and metallicites from the SED fitting for JADES-GS-z14-0 (*M*_★_ ≲ 10^9^ *M*_⊙_ and *Z*_★_ ≲ 10% *Z*_⊙_), we assume the lower value of the gas-phase metallicity to break the double-valued degeneracy between [O iii]/Hβ. The inferred stellar masses and metallicities for the individual galaxies that have Hβ line flux measurements from JADES DR3^32^ are similar to those derived for JADES-GS-z14-0, so we can make the same assumption for those galaxies. Thus, given that the line ratio [O iii]/Hβ for JADES-GS-z14-0 is smaller than typical values observed for galaxies at *z* ≈ 8, these results suggest a smaller gas-phase metallicity for JADES-GS-z14-0 when compared with galaxies at *z* ≈ 8.

## Figures and Tables

**Extended Data Table 1:**
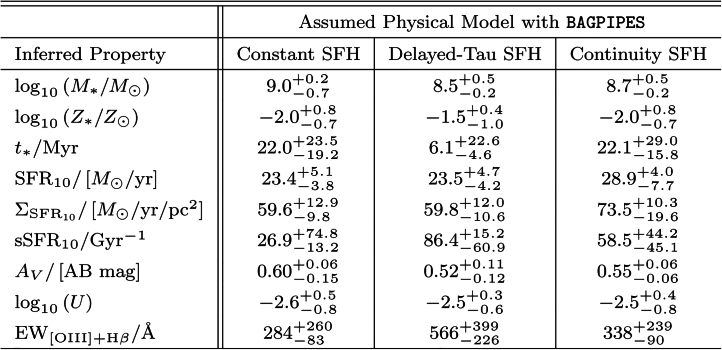
Inferred physical properties of JADES-GS-z14-0 from SED modeling with BAGPIPES

Columns: (1) name and units of the inferred property, (2) measurements using the constant SFH model, (3) measurements using the delayed-tau SFH model, and (4) measurements using the continuity SFH model. Reported measurements are median values and 1*σ* confidence intervals.

**Extended Data Table 2:**
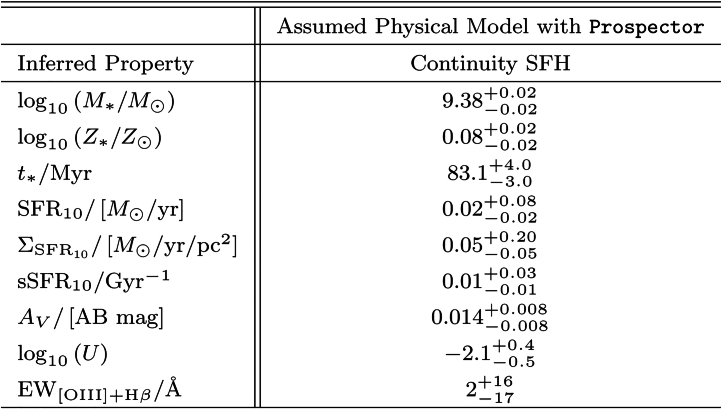
Inferred physical properties of JADES-GS-z14-0 from SED modeling with Prospector

Columns: (1) name and units of the inferred property and (2) measurements using the continuity SFH model. Reported measurements are median values and 1*σ* confidence intervals.

## Data Availability

The NIRCam data that support the findings of this study are publicly available at https://archive.stsci.edu/hlsp/jades. The MIRI data that support the findings of this study will be made available in a future release; advanced access may be granted on reasonable request to the corresponding author.

## Code availability

The AstroPy^72,73^ software suite is publicly available, as is BAGPIPES^16^, Cloudy^57^, dynesty^59^, ForcePho (B.D.J. et al., manuscript in preparation), FSPS^60,61^, GALFIT^14,15^, Matplotlib^74^, nautilus^50^, NumPy^75,76^, Pandas^77^, photutils^78^, Prospector^17^, python-fsps^62^, SciPy^79^, seaborn^80^, Tiny Tim^81^ and WebbPSF^82^.
